# Protein Phosphorylation Changes During Systemic Acquired Resistance in *Arabidopsis thaliana*

**DOI:** 10.3389/fpls.2021.748287

**Published:** 2021-11-11

**Authors:** Qingfeng Zhou, Qi Meng, Xiaomin Tan, Wei Ding, Kang Ma, Ziqin Xu, Xuan Huang, Hang Gao

**Affiliations:** ^1^College of Biology and Food, Shangqiu Normal University, Shangqiu, China; ^2^Key Laboratory of Resource Biology and Biotechnology in Western China, Ministry of Education, Provincial Key Laboratory of Biotechnology, College of Life Sciences, Northwest University, Xi’an, China; ^3^Shanghai Omicsspace Biotechnology Co., Ltd., Shanghai, China

**Keywords:** phosphoproteomics, *Arabidopsis*, systemic acquired resistance, data-independent acquisition, calcium-dependent protein kinase (CDPK), MAPK

## Abstract

Systemic acquired resistance (SAR) in plants is a defense response that provides resistance against a wide range of pathogens at the whole-plant level following primary infection. Although the molecular mechanisms of SAR have been extensively studied in recent years, the role of phosphorylation that occurs in systemic leaves of SAR-induced plants is poorly understood. We used a data-independent acquisition (DIA) phosphoproteomics platform based on high-resolution mass spectrometry in an *Arabidopsis thaliana* model to identify phosphoproteins related to SAR establishment. A total of 8011 phosphorylation sites from 3234 proteins were identified in systemic leaves of *Pseudomonas syringae* pv. *maculicola* ES4326 (*Psm* ES4326) and mock locally inoculated plants. A total of 859 significantly changed phosphoproteins from 1119 significantly changed phosphopeptides were detected in systemic leaves of *Psm* ES4326 locally inoculated plants, including numerous transcription factors and kinases. A variety of defense response-related proteins were found to be differentially phosphorylated in systemic leaves of *Psm* ES4326 locally inoculated leaves, suggesting that these proteins may be functionally involved in SAR through phosphorylation or dephosphorylation. Significantly changed phosphoproteins were enriched mainly in categories related to response to abscisic acid, regulation of stomatal movement, plant–pathogen interaction, MAPK signaling pathway, purine metabolism, photosynthesis-antenna proteins, and flavonoid biosynthesis. A total of 28 proteins were regulated at both protein and phosphorylation levels during SAR. RT-qPCR analysis revealed that changes in phosphorylation levels of proteins during SAR did not result from changes in transcript abundance. This study provides comprehensive details of key phosphoproteins associated with SAR, which will facilitate further research on the molecular mechanisms of SAR.

## Introduction

Plants have evolved multi-layered defense systems that utilize constitutive, inducible defense strategies to combat pathogens (Shah and Zeier, 2013). In pathogen-infected tissues, conserved molecules or structures of the pathogens are recognized by plant pattern recognition receptors (PRRs), leading to activation of defense responses; this defensive process is termed pathogen-associated molecular pattern (PAMP)-triggered immunity (PTI) (Naveed et al., 2020). On the other hand, during the course of coevolution, pathogens have developed effector proteins that are released inside host cells, and interfere with PTI. Plants recognize such effector proteins through resistance (R) genes and consequently activate stronger, longer-term defense responses and hypersensitive cell death reaction, collectively termed effector-triggered immunity (ETI) (Naveed et al., 2020). Besides these mechanisms which occur in pathogen-infected tissues, plants are able to initiate defense responses in tissues distant from the infection site, thereby enhancing the resistance of the systemic, bacteria-free tissues to subsequent pathogen challenge (Cameron et al., 2010); this phenomenon is termed systemic acquired resistance (SAR) (Kachroo and Kachroo, 2020).

Induction of SAR depends on mobile signals (inducers) generated in pathogen-infected tissues. Methyl salicylate (MeSA), the methyl ester of salicylic acid (SA), was the first confirmed mobile signal of SAR (Park et al., 2010), and its discovery was followed rapidly by reports of many others, including azelaic acid (AzA), glycerol-3-phosphate (G3P), pipecolic acid (Pip), N-hydroxy-pipecolic acid (NHP), dehydroabietinal (DA), nicotinamide adenine dinucleotide (NAD), and nicotinamide adenine dinucleotide phosphate (NADP) (Maldonado et al., 2002; Jung et al., 2009; Chanda et al., 2011; Chaturvedi et al., 2012; Návarová et al., 2012; Chen et al., 2018; Hartmann et al., 2018; Wang C. et al., 2019). Certain volatile organic compounds (e.g., α-pinene, β-pinene, and camphene) can also promote SAR and induce defense responses in neighboring plants (Riedlmeier et al., 2017). NHP is synthesized via hydroxylation of Pip by flavin-dependent monooxygenase 1 (FMO1) (Chen et al., 2018; Hartmann et al., 2018; Hartmann and Zeier, 2019). A series of studies have provided evidence that NHP plays a critical role in SAR induction in different plant species (Holmes et al., 2019; Schnake et al., 2020). Recent research illustrates that UDP-dependent glycosyltransferase 76B1 (UGT76B1) inactivates NHP by forming the inactive NHP-N-O-glucoside (NHPG) to rationally control the growth-immunity trade-off in crop plants (Bauer et al., 2021; Cai et al., 2021; Holmes et al., 2021; Mohnike et al., 2021; Zeier, 2021).

Phosphorylation is an important post-translational modification (PTM) of proteins (Li et al., 2015). Kinase-mediated covalent addition of a phosphate group to tyrosine, threonine, or serine residues in eukaryotes, and to other amino acids (aspartate, lysine, histidine, glutamate, and arginine) in prokaryotes, followed by removal of the phosphate group by a protein phosphatase, is the basis of essential signaling and regulatory mechanisms in these organisms (Batalha et al., 2012; Li et al., 2015). Protein phosphorylation plays key roles in numerous signal transduction pathways by modulating activity, stability, and localization of proteins, as well as protein–protein interactions (Zhang et al., 2017). At a given time in any eukaryotic cell, roughly one-third of proteins are phosphorylated, and phosphorylated and non-phosphorylated forms of a given protein often coexist (Qeli and Ahrens, 2010; Zhang et al., 2017). Many specific kinases have been shown to play essential roles in defense responses to pathogen invasion. Elucidation of phosphorylation events that occur in biotic stress responses will increase our understanding of plant physiological processes (Stecker et al., 2014).

Phosphoproteomics technology based on high-precision mass spectrometry (MS) platforms has been used extensively as a research tool for the characterization of phosphorylated components in proteomes (Noujaim et al., 2016). Phosphoproteomics data have been collected primarily by data-dependent acquisition (DDA) methods (Chapman et al., 2014). In this approach, the most abundant ionized species from each precursor ion scan are selected for subsequent isolation, activation, and tandem mass analysis (Courchesne et al., 1998). Irreproducibility and imprecision play fundamental roles in DDA methods; if too many peptide species co-elute and appear in a single MS1 scan, only the most abundant peptides are stochastically sampled, and the rest are missed (Hu et al., 2016). Data-independent acquisition (DIA) is an alternative method used increasingly during the past decade because of its superior detection and quantitation capability. In DIA, all ionized species within selected *m/z* windows are subjected to subsequent tandem mass analysis (Bruderer et al., 2015; Li et al., 2020). This approach provides a broader dynamic range of detected signals, and better reproducibility (for identification), sensitivity, and accuracy (for quantification) in comparison with DDA (Bilbao et al., 2015).

Many studies have revealed alterations of protein metabolism and transcription in systemic tissues of plants following SAR induction (Gruner et al., 2013; Bernsdorff et al., 2016; Wang et al., 2016; Schwachtje et al., 2018; Kumar et al., 2020). Dynamic phosphorylation events during PTI and ETI are also investigated (Kadota et al., 2019; Zhang et al., 2019). However, little is known regarding changes of phosphoproteins in systemic leaves of SAR-induced plants. The purpose of the present study was to identify significantly altered phosphoproteins in systemic tissues of locally pathogen-inoculated plants. We induced SAR in an *Arabidopsis thaliana* model using virulent *Pseudomonas syringae* pv. *maculicola* ES4326 (*Psm* ES4326), and used DIA phosphoproteomic analysis based on high-precision MS to identify altered phosphoproteins. Significantly changed phosphorylation levels were observed for proteins involved in a variety of biological processes/pathways, including plant–pathogen interactions, amino acid metabolism, photosynthesis, mitogen-activated protein kinase (MAPK) signaling, and secondary metabolism. Numerous phosphoproteins were identified as potential components in SAR.

## Materials and Methods

### Plant Materials and Growth Conditions

Four-week-old wild-type (WT) *A. thaliana* plants (Col-0 background) were placed in individual pots containing perlite, vermiculite, and nutrient soil (1:1:1, v/v/v), and grown in a chamber [22°C, 16 h light (photon flux density 70 μmol m^–2^ s^–1^)/8 h dark cycle, relative humidity 65%].

### Bacteria Culture

Virulent *P. syringae* pv *maculicola* strain ES4326 (*Psm*) was grown in King’s B medium containing 50 mg/L streptomycin at 28°C. Overnight log-phase cultures with OD600 = 0.8–1.0 were centrifuged at 5000 rev/m for 5 min, washed 3× with 10 mM MgCl_2_, and resuspended with 10 mmol/L MgCl_2_ to OD600 = 0.002 for leaf inoculation.

### Induction and Assessment of Systemic Acquired Resistance

Compatible virulent *Psm* ES4326 is capable of inducing full SAR in *Arabidopsis* within 2 days (Návarová et al., 2012; Bernsdorff et al., 2016). For each plant, three local leaves of 4-week-old WT were infiltrated with a suspension of virulent *Psm* ES4326 (V) at OD600 = 0.002 to induce SAR. 10 mM MgCl_2_ was used for the control treatment. Systemic leaves (termed SL, typically leaf 7–9) of *Psm* ES4326 or MgCl_2_ inoculated plants (termed SL-V and SL-CK) were harvested at 48 h for DIA phosphoproteomic analysis.

For bacterial growth assay, leaves were secondarily inoculated with *Psm* ES4326 (OD600 = 0.002) after local treatment. After 3 days, the growth of bacteria in leaves was scored by homogenizing disks taken from infiltrated areas of three different leaves, as described by Carella et al. (2016).

### Quantitative Real-Time Polymerase Chain Reaction (PCR)

Total RNA was isolated from frozen leaves using RNAiso Reagent (TaKaRa Bio, Otsu, Japan) as per the manufacturer’s instructions. mRNA was reverse-transcribed to cDNA with oligo(dT) primers and reverse transcriptase (Omniscript RT Kit; Roche) as per the manufacturer’s instructions; 1 μL cDNA and 5 μL SYBR Green (Roche) were used in PCR reactions of total reaction volume 10 μL containing 0.75 μM gene-specific primers. Quantitative real-time PCR (RT-qPCR) was performed using 96-well CFX96 Real-Time System (Bio-Rad, Hercules, CA, United States). Data were normalized using *ACTIN8* (GenBank accession # AY870652.1) as reference gene. Primers used are listed in Supplementary Table 12. Relative expression levels were calculated by 2^–ΔΔCt^ (cycle threshold) method.

### Total Protein Extraction

Total proteins were extracted by urea extraction method (Hou et al., 2015), using three individual biological replicates for each treatment. Briefly, 1 g of leaf tissue was weighed, homogenized by grinding in liquid nitrogen, lysed with lysis buffer (Tris–HCl [pH 8], 8 M urea, 0.2% SDS, 1× phosphoprotein protease inhibitor complex), ultrasonicated on ice for 5 min, and centrifuged (12,000 × *g*, 20 min, 4°C). The supernatant was transferred to a clean tube. Proteins were precipitated in pre-cooled acetone at −20°C for 2 h, washed 2× in 75% ethanol, and resolved in lysis buffer. Protein concentration was quantified by Bradford assay.

### Protein Digestion

Proteins from each sample were reduced with 2 mM dithiothreitol for 1 h at 56°C, alkylated with 20 mM iodoacetic acid for 1 h at room temperature in the dark, and digested for 16 h on a 30-kDa filter unit (Millipore) with trypsin (Promega) at enzyme-to-substrate ratio 1:50 at 37°C. Part of peptides from samples were mixed equally. The mixture peptides (mix-sample) and the remaining peptides (single-sample) were all desalted with C18 cartridge to reduce urea concentration. The mixture peptides were used for library construction, and the peptides from the single-sample were used for DIA analysis.

### Phosphopeptides Enrichment: Immobilized Metal Affinity Chromatography (IMAC)

Phosphopeptides enrichment was performed as previously described (Zhou et al., 2011). Briefly, Peptides (including mixture peptides) were dissolved with binding buffer, then incubated with IMAC beads (Sigma-Aldrich) for 2 h at room temperature. Samples were loaded 3× in a constricted GELoader tip and washed 4× with 30% acetonitrile/250 mM ethanol. Phosphopeptides were eluted using 50 mM Na_2_HPO_4_/NH_3_ (pH 10.0), desalted, and subjected to LC-MS/MS analysis.

### Library Construction

Phosphopeptides from the mix-sample were fractionated using a C18 column (Waters BEH C18 4.6 × 250 mm, 5 μm) on Rigol L3000 HPLC. Mobile phases A (2% acetonitrile, PH 10.0) and B (98% acetonitrile, pH 10.0) were used to develop a gradient elution. The solvent gradient was set as follows: 3% B, 5 min; 3–8% B, 0.1 min; 8–18% B, 11.9 min; 18–32% B, 11 min; 32–45% B, 7 min; 45–80% B, 3 min; 80% B, 5 min; 80–5%, 0.1 min; 5% B, 6.9 min. The eluates were monitored at UV 214 nm. Collected fractions were combined into six pooled fractions, each of which was concentrated by vacuum centrifugation and reconstituted in 0.1% (v/v) formic acid in water. Then, peptide concentration was determined at OD_280_. For transition library construction, shotgun proteomic analyses were performed using EASY-nLCTM 1200 UHPLC system coupled with Orbitrap Fusion Lumos Mass Spectrometer (Thermo Fisher) operating in DDA mode; 2 μg total peptides from each fraction sample were separated on ReproSil-Pur 120 C18-AQ analytical column (15 cm × 150 μm, 1.9 μm), using 120 min linear gradient from accomplished using a segmented 2 h gradient from 5 to 28% Solvent B (0.1% formic acid in 100% ACN) for 90 min, followed by 28–35% Solvent B for 10 min, 35–90% Solvent B for 2 min, and then 90% Solvent B for 18 min at a flow rate of 600 nL/min, buffer A (0.1% FA in H_2_O). The 40 most abundant precursor ions from full MS scan were selected for fragmentation and higher-energy collisional dissociation (HCD) fragment analysis. Single-sample peptides were injected into the coupled system as above operating in DIA mode, with liquid conditions as above. For DIA acquisition, MS1 and MS2 resolution were set, respectively, to 60,000 and 30,000, at 200 *m/z*. *m/z* range was from 350 to 1500, with variable 60 cycles (Supplementary Table 1), full scan AGC target 3 × 10^6^, and IT 50 ms. DIA settings were NCE 27%, target value 1 × 10^6^, and maximum injection time set to auto, such that MS operated continuously in parallel ion filling and detection mode.

### Data Analysis

Data-dependent acquisition and DIA data were analyzed using software programs Proteome Discoverer V. 2.4 (Thermo Fisher), Biognosys Spectronaut V. 9.0, and R statistical framework. DDA MS raw files were analyzed using Proteome Discoverer, and peak lists were searched against UniProt protein database (*A. thaliana*), with mass tolerance set to 10 ppm for precursor and 10 ppm for fragment. Methionine oxidation (M), acetyl (Protein-N term), and phospho (S/T/Y) were set as variable modifications and cysteine carbamidomethylation as fixed modification. False discovery rate (FDR) was set to 1% for proteins and peptides, to screen out reliable phosphopeptides. MS1-based label-free quantification (LFQ) was performed using MaxLFQ algorithm (Cox et al., 2014).

Mass spectrometry 2-based LFQ was performed by analyzing DIA raw data with Biognosys Spectronaut. Data were analyzed as described by Gotti et al. (2021). Briefly, DIA files were processed using Spectronaut with default settings, with PTM localization activated and site confidence score cutoff set to 0.75 (for spectral library analysis) data filtering set to *Q*-value and Normalization Strategy set to Global Normalization (new default): choose to use the median; In Spectronaut, two normalization strategies are available: Central Tendency Normalization (Global Normalization) and Local Regression Normalization (Local Normalization). Central Tendency Normalization centers peptide abundance ratios around a median, mean, or a constant to adjust for the effects of independent systematic bias.

Peptides were considered truly phosphorylated if they were phosphorylated in two or three of the three biological replicates. Differences with phosphopeptide abundance fold change ≥1.5 and *p* ≤ 0.05 (Student’s *t*-test) were considered statistically significant. Protein function annotation was performed using the software program Blast2GO (V. 2.7.2), and pathways were annotated using an online tool KEGG Mapper^1^.

## Results

### Characterization of Phosphoproteome

**FIGURE 1:**
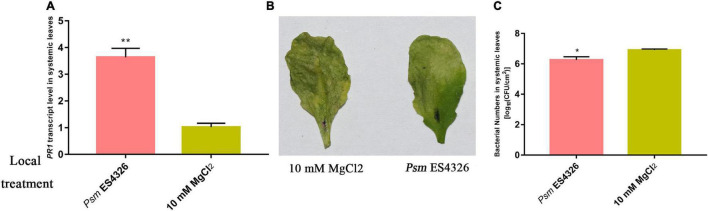
Investigation of SAR. Col-0 plants were inoculated with virulent *Psm* ES4326 or 10 mM MgCl_2_. **(A)** Expression analysis of *PR1* in systemic leaves of *Psm* ES4326-inoculated plants. Phenotype **(B)** and bacterial growth assay **(C)** of systemic leaves following secondary infection with *Psm* ES4326, after the initial infection of local leaves with *Psm* ES4326 or MgCl_2_. *p < 0.05 and **p < 0.05 Data are presented as mean ± SE (*n* = 3 or 4).

**FIGURE 2:**
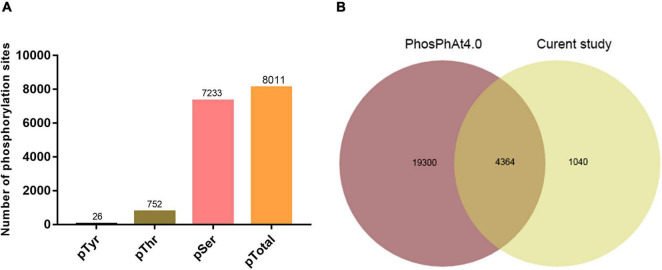
Data-independent acquisition (DIA) phosphoproteomic analysis. **(A)** Distribution of phosphosite types. **(B)** Comparison of phosphopeptides identified in this study with *Arabidopsis* phosphorylation database (PhosPhat 4.0).

Successful SAR induction was confirmed by RT-qPCR analysis of *pathogenesis-related gene 1* (*PR1*), phenotypic observation, and bacterial growth assay (Figure 1). Leaves of experimental and control plants were collected, and phosphorylated protein profiles were analyzed using the phosphoproteomics platform. A total of 6559 unique phosphopeptides were detected and assigned to 3234 proteins with 8011 detected phosphorylation sites (Figure 2A and Supplementary Table 2). Comparison with known phosphopeptides in the genus revealed that 1040 phosphopeptides in our dataset were not present in the *Arabidopsis* Protein Phosphorylation Site Database (PhosPhAt) 4.0^2^ (Figure 2B; Bernsdorff et al., 2016). Our findings thus enlarge the *Arabidopsis* Protein Phosphorylation DataBase and also provide a basis for functional studies of proteins that undergo phosphorylation modification during SAR. Among the 8011 identified phosphorylation sites, 7233 involve serine (pSer), 752 threonine (pThr), and 26 tyrosine (pTyr) (Figure 2A). We found 26 pTyr-containing proteins with 26 pTyr sites (Supplementary Table 3), which is consistent with the low frequency of Tyr phosphorylation in plants.

### Phosphoproteins That Undergo Significant Changes in Systemic Leaves of Systemic Acquired Resistance-Induced Plants

**FIGURE 3:**
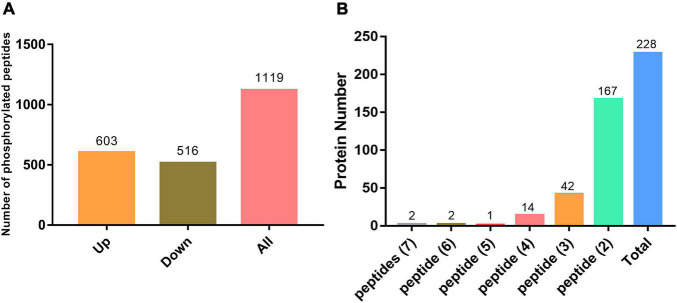
Phosphoproteomic analysis. **(A)** Significantly changed phosphorylated peptides in systemic leaves of *Psm* ES4326-inoculated plants. **(B)** Phosphoproteins containing three or more significantly changed phosphorylated peptides in systemic leaves of *Psm* ES4326-inoculated plants.

We performed pairwise comparisons of phosphopeptide abundance in systemic leaves of *Psm* ES4326 vs. mock (MgCl_2_) locally inoculated plants. Phosphopeptides with quantified intensities in at least two replicates were used for further analysis; 336 and 216 unique phosphopeptides were only detected in *Psm* ES4326- and mock-treated samples, respectively. In addition, statistical analysis determined 567 significantly changed phosphopeptides (fold change >1.5 or <0.67, *p* < 0.05): 267 that were found at higher abundance and 300 that were found at lower abundance upon *Psm* ES4326 treatment compared with mock treatment (Supplementary Table 2). Phosphopeptides detected only in *Psm* ES4326-treated plants, or only in mock-treated plants, were assigned, respectively, to “upregulated” or “downregulated” group. Compared with MgCl_2_ treatment group, 1119 phosphopeptides (603 upregulated and 516 downregulated) from 866 proteins showed significantly altered abundance in systemic leaves of *Psm* ES4326 locally treated plants (Figure 3A and Supplementary Table 2). Among the 866 proteins, 228 had two or more significantly changed phosphopeptides (Figure 3B and Supplementary Table 4), and five had five or more significantly changed phosphopeptides; i.e., ATP-binding cassette (ABC) transporter g family member 36 (ABCG36), IQ-domain 14 (IQD14), eukaryotic translation initiation factor isoform 4G-1 (EIF4G1), TSS (REDUCED CHLOROPLAST COVERAGE 2), and AT5G04550. These five proteins are presumably subject to complex phosphorylation modification regulation in systemic leaves of *Psm* ES4326 locally inoculated plants.

**FIGURE 4:**
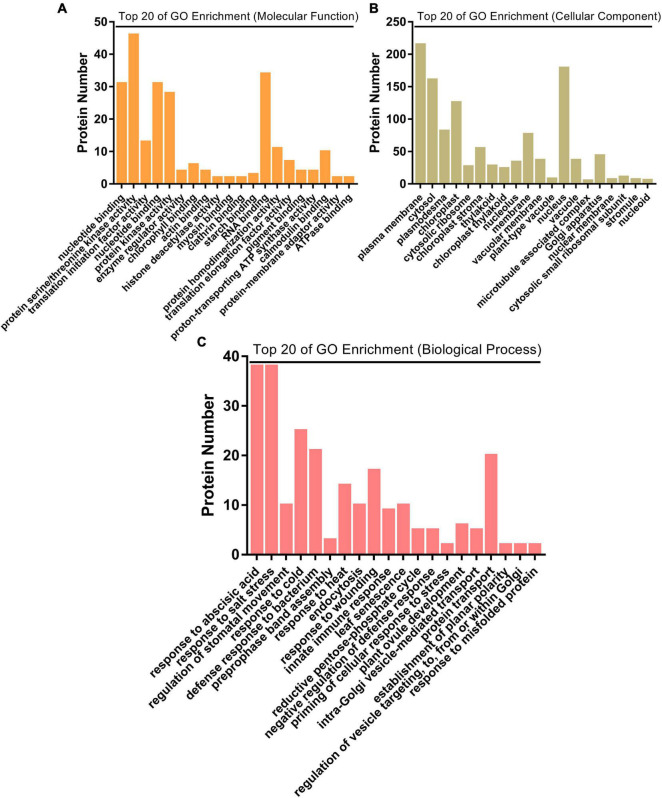
GO enrichment analysis of significantly changed phosphoproteins. **(A)** Top 20 of GO enrichment analysis on molecular function. **(B)** Top 20 of GO enrichment analysis on cellular component. **(C)** Top 20 of GO enrichment analysis on biological process.

**FIGURE 5:**
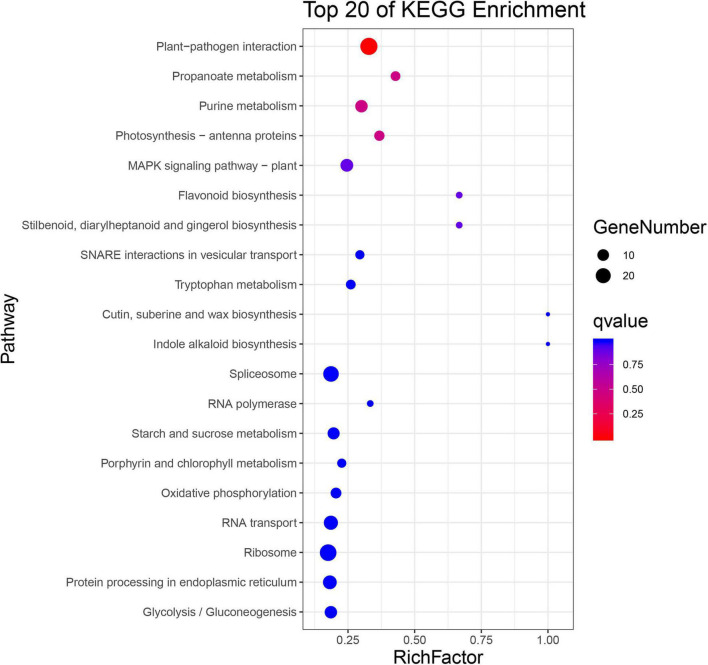
KEGG enrichment analysis of significantly changed phosphoproteins.

Biological functions of the significantly changed phosphoproteins were investigated by GO and KEGG enrichment analysis. GO enrichment analysis was conducted on the data set in the context of biological process (BP), molecular function (MF), and cellular component (CC) (Figure 4). Molecular functions were enriched in nucleotide binding, protein serine/threonine kinase activity, translation initiation factor activity, protein kinase activity, and enzyme regulator activity (Figure 4A). As to the subcellular localization, plasma membrane, cytosol, plasmodesma, chloroplast, and cytosolic ribosome were enriched (Figure 4C). For biological process, the significantly changed phosphoproteins were mainly enriched in a variety of processes, including response to abscisic acid (ABA), regulation of stomatal movement, defense response to bacterium, response to wounding, and innate immune response (Figure 4C). They showed enhanced levels in pathways involved in plant–pathogen interaction, purine metabolism, photosynthesis-antenna proteins, MAPK signaling pathway, flavonoid biosynthesis, cutin biosynthesis, and indole alkaloid biosynthesis (Figure 5), suggesting association of these pathways with SAR establishment.

### Screening of Phosphoproteins Associated With Systemic Acquired Resistance Establishment

**FIGURE 6:**
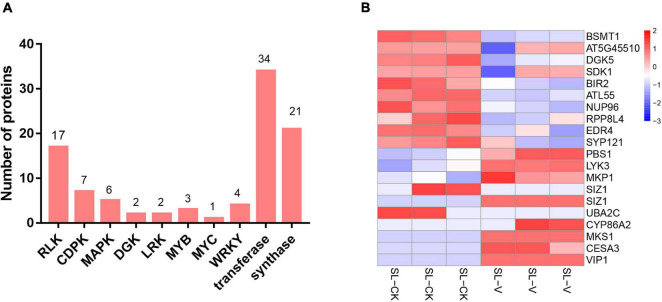
Overview of significantly changed kinases, TFs, transferases, synthases, and defense response-related proteins identified in phosphoproteome. **(A)** Numbers of significantly changed kinases, TFs, transferases, and synthases. **(B)** Heat map of significantly changed defense response-related phosphopeptides from systemic leaves of *Psm* ES4326 and mock locally inoculated plants. Data were normalized based on Z-score, and hierarchical analysis was performed by bottom-up clustering method using Euclidean distance. V, virulent *Psm* ES4326; CK, 10 mM MgCl_2_; SL-V, systemic leaves of *Psm* ES4326 locally inoculated plants; SL-CK, systemic leaves of MgCl_2_ locally inoculated plants.

Protein kinases and transcription factors (TFs) play numerous essential roles in plants (Ganguly et al., 2012; Ye et al., 2015). The 866 significantly changed proteins mentioned in the preceding section include 43 TFs (Supplementary Table 5) and 84 kinases (Supplementary Table 6), consisting of 17 receptor-like kinases (RLKs), seven calcium-dependent protein kinases (CDPKs), six MAPKs (Supplementary Table 8), three MYB TFs, one MYC TF, and four WRKY TFs (Figure 6A). Nineteen proteins involved in defense responses showed significantly changed phosphopeptide abundances in systemic leaves of *Psm* ES4326 locally inoculated plants (Figure 6B and Supplementary Table 7); these include salicylate/benzoate carboxyl methyltransferase (BSMT1), diacylglycerol kinase 5 (DGK5), SDK1 (receptor kinase 1), BAK1-interacting receptor-like kinase 2 (BIR2), NUP96 (suppressor of auxin resistance 3), and enhanced disease resistance 4 (EDR4). 23 abiotic stress-related proteins showed differential phosphorylation (Supplementary Table 8), suggesting that they are also functionally involved in SAR establishment.

**FIGURE 7:**
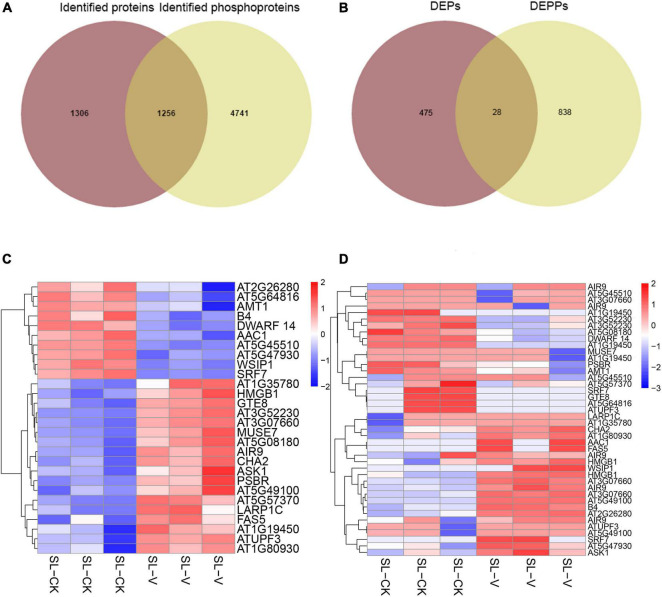
Comparative analyses of proteome and phosphoproteome. **(A)** Overlap of identified phosphoproteins and identified proteins. **(B)** Overlap of significantly changed proteins and significantly changed phosphoproteins. **(C)** Heat map of significantly changed proteins from systemic leaves of *Psm* ES4326 and mock locally inoculated plants. **(D)** Heat map of significantly changed phosphopeptides from systemic leaves of *Psm* ES4326 and mock locally inoculated plants. DEPs, differentially expressed proteins; DEPPs, differentially expressed phosphoproteins. Analytical procedures are as described in Figure 6B.

One thousand two hundred and fifty-six proteins were identified from both our proteomic data (not shown) and phosphoproteomic data (Figure 7A). Further analysis of significantly changed phosphoproteins relative to the quantified proteome showed that only 28 proteins had significantly changed abundance at both protein level and phosphoprotein level (Figures 7B–D); these included AT5G45110 [leucine-rich repeat (LRR) family protein], WUS-interacting protein 1 (WSIP1), auxin-induced in root cultures 9 (AIR9), AT5G64816 (thionin-like gene), and arabidopsis serine/threonine kinase 1 (ASK1). These findings indicate that abundance changes of most proteins do not strongly affect their phosphorylation level during SAR establishment.

**FIGURE 8:**
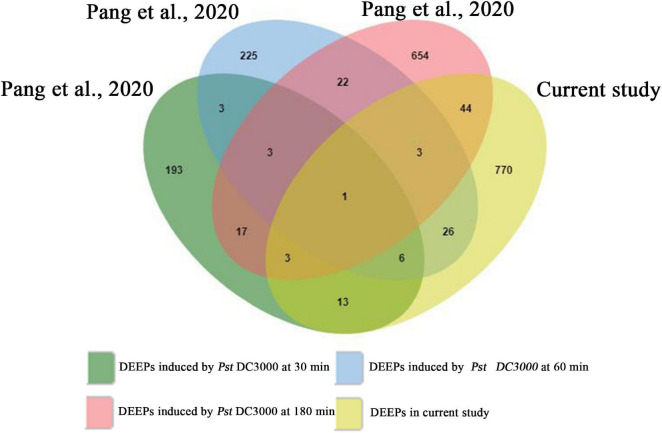
Venn diagram showing the overlapping phosphoproteins from previously published stomatal immune response-related phosphoproteomics and the present study. DEEPs, differentially expressed phosphoproteins.

To assess whether SAR-related phosphorylated proteins in systemic leaves are part of a more general biotic response, we compared our data set with previously published stomatal immune response-related phosphoproteome data set (Figure 8; Pang et al., 2020). We found 23, 46, and 51 proteins were regulated by phosphorylation/dephosphorylation both in stomata infected by *Pst* DC3000 at different times (30, 60, and 180 min, respectively) and in systemic leaves of *Psm* ES4326 locally inoculated plants (Supplementary Table 9). Interestingly, one protein, ABCG11 was detected in all data sets, possibly indicating a general role in biotic stress responses.

### Transcriptional Analysis of Significantly Changed Phosphoproteins

**FIGURE 9:**
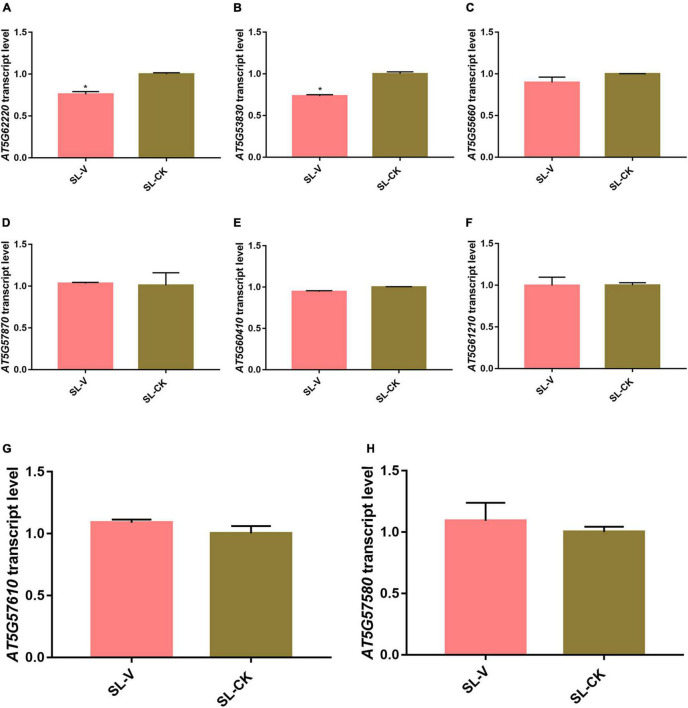
Quantitative real-time PCR (RT-qPCR) analysis of mRNA abundance of significantly changed phosphoprotein genes. **(A–H)** mRNA abundance of *AT5G62220* **(A)**, *AT5G53830* **(B)**, *AT5G55660* **(C)**, *AT5G57870* **(D)**, *AT5G60410* **(E)**, *AT5G61210* **(F)**, *AT5G57610* **(G)**, and *AT5G57580* **(H)** in systemic leaves of Psm ES4326- and Mock- locally inoculated plants. Expression was normalized relative to ACTIN8. Data are presented as mean ± SE (*n* = 3). *Represent *p* < 0.05 (*t*-test).

Previous studies have shown that the changes at the protein and phosphoprotein levels are not directly regulated at the transcription level in pathogens infected tissues (Hou et al., 2015; Zhang et al., 2018; Pang et al., 2020). To investigate the correlation of the mRNA transcript abundance with the protein phosphorylation intensity level during SAR, expression of significantly changed phosphoproteins at the mRNA level was evaluated by randomly selecting eight of them and determining their relative gene transcription levels by RT-qPCR. Transcription levels of the six genes other than *AT5G62220* and *AT5G53830* did not differ notably in leaves of *Psm* ES4326- vs. mock-inoculated plants (Figure 9). Meanwhile, the abundance changes of most proteins do not strongly affect their phosphorylation level during SAR establishment (Figures 7A,B). These results suggest that alterations of protein phosphorylation levels during SAR was majorly due to the occurrence of phosphorylation event in the pre-existing proteins, instead of the quantity change caused by protein synthesis or degradation.

## Discussion

Systemic acquired resistance in plants confers long-lasting resistance against a broad spectrum of pathogens (Durrant and Dong, 2004). SAR induction is a complex regulatory process (Chen J. et al., 2019). Signaling mechanisms of SAR have been intensively studied during the past decade (Howden et al., 2017; David et al., 2019), but the molecular mechanism of SAR induction remains poorly known. We investigated phosphorylation events related to SAR establishment in *Arabidopsis*, using DIA phosphoproteomic analysis based on high-precision MS. We identified 8011 non-redundant phosphopeptides from 3234 *Arabidopsis* phosphoproteins present at initial SAR establishment and found that 866 proteins showed significantly changed phosphorylation levels during SAR. This study has two outcomes: (1) an improved knowledge of signaling networks and defense responses through direct correlation of phosphoprotein changes with SAR and (2) identification of numerous candidate phosphoproteins related to SAR that can be mined by others to address their additional important questions.

### Hormone Signaling

Salicylic acid is a critical plant defense hormone that promotes immunity against biotrophic and semibiotrophic pathogens. It plays essential roles in basal defense and the amplification of immune signals in systemic tissues during SAR (Ding et al., 2016). Consistently, a plenty of proteins related to SA mediated signaling pathway showed significantly changed phosphorylation levels in systemic leaves (Supplementary Table 10).

GO enrichment analysis on biological processes showed that term of response to ABA and regulation of stomatal movement are the two most significant biological processes (Figure 4C), suggesting that ABA and stomatal movement may be also involved in SAR establishment. ABA is a sesquiterpene signaling molecule produced in all kingdoms of life. In plants, ABA is best known as a phytohormone regulating abiotic stress responses (Lievens et al., 2017). ABA acts antagonistically against the SA-mediated immune signaling pathway (Jiang et al., 2010). Non-expressor of pathogenesis-related genes 1 (NPR1) is the key regulator of plant local and systemic immunity. In *A. thaliana*, SA regulates NPR1 activity by sophisticated mechanisms: (1) SA regulates NPR1 protein subcellular localization by inducing cellular redox changes (Mou et al., 2003); (2) SA activates NPR1 by changing NPR1 conformation, leading to transcription of SA-dependent genes (Ding et al., 2016); and (3) SA regulates NPR1 activity by inducing its phosphorylation (Spoel et al., 2009). Ding et al. (2016) reported that SA and ABA antagonistically regulate cellular NPR1 protein homeostasis by modulating its degradation. Sequential SA and ABA signaling is correlated with dynamic changes in NPR1 protein levels. ABA signaling is required for full-scale induction of SA- and NPR1-dependent genes (Ding et al., 2016). The content of ABA both increased in uninfected halves (adjacent tissues) of pathogens infected tissues and stomata in systemic leaves of SAR-induced plants (Ding et al., 2016; David et al., 2020). In our present work, GO enrichment analysis on biological processes showed that term of response to ABA was significantly enriched (Figure 4C and Supplementary Table 10), suggesting that ABA may be also involved in SAR establishment by regulating phosphorylation/dephosphorylation of these proteins in systemic leaves.

### Proteins Related to Regulate Stomatal Movement

Stomata are the passive entry point for many plant pathogens. Plants close stomata when they sense attack to restrict pathogen entry. Stomata closure is regulated by phytohormones ABA, SA, and JA in pathogen-infected tissues, which forms part of the plant immune response that is commonly referred to as PTI (Geng et al., 2016; Dutton et al., 2019; Xiang et al., 2021). Like SA, the content of ABA also increases in uninfected halves of pathogens infected tissues and in systemic stomatal of SAR-induced plants (Ding et al., 2016; David et al., 2020). David et al. (2019) suggested that stomata in systemic tissues may also play additional roles in preventing future infections as a part of the overall plant immunity. Consistent with this, GO enrichment analysis of significantly changed phosphoproteins showed that the term of regulation of stomatal movement was significantly enriched (Figure 4C). These proteins include open stomata 2 (OST2), thioglucoside glucohydrolase 1 (TGG1), TGG2, ABA-responsive kinase substrate 1 (AKS1), and glycine-rich protein 7 (GRP7) (Supplementary Table 10). TGG, the β-thioglucoside glucohydrolase enzyme, is a highly abundant protein present in crucifer plants and catalyzes degradation of glucosinolates to produce isothiocyanates. The glucosinolate-myrosinase system plays important role in plant resistance to bacteria and insects (Rhaman et al., 2020). In *Arabidopsis*, TGG1 and TGG2 redundantly regulate stomata closure downstream of ROS production and upstream of cytosolic Ca^2+^ elevation in ABA- and MeJA-mediated signaling pathway (Islam et al., 2009). Yamauchi et al. (2016) found that OST2 plays a major role in blue light-dependent stomatal opening in *Arabidopsis*. Whether these proteins could regulate systemic stomata closure by phosphorylation/dephosphorylation during SAR require further study.

### Proteins Regulated by Complex Phosphorylation Modification During Systemic Acquired Resistance Establishment

Protein phosphorylation is involved in all key regulatory processes that mediate plant growth/development and stress responses. Phosphoproteins play functional roles in stress responses and provide the basic backbones of complex signaling networks (Yin et al., 2018). In our model system, 228 proteins were found to have two or more significantly changed phosphopeptides (Figure 3B and Supplementary Table 4). Among these, five proteins (ABCG36, IQD14, EIF4G1, TSS, and AT5G04550) had five or more significantly changed phosphopeptides. Functions of these proteins are evidently regulated by complex phosphorylation modification during SAR induction.

ABC transporters clearly play essential roles in plant growth/development and biotic/abiotic stress responses (Campe et al., 2016). Little is known regarding whether or how activities of these transporters are mediated by protein modification in plant resistance to biological stresses. We observed significantly changed phosphorylation levels for seven ABC transporters [ABCC4, ABCB6, ABCG11, ABCG12, ABCC14, ABCG19, and ABCG36 (PEN3)] in systemic leaves of *Psm* ES4326 locally inoculated plants (Supplementary Table 11), indicating functional involvement of these proteins in SAR establishment. ABC transporters transport a wide variety of substrates, including toxic compounds, secondary metabolites, inorganic acid, and drugs (Martinoia et al., 2002; Verrier et al., 2008). ABCG36 is localized in the plasma membrane in plant cells and is necessary for penetration resistance against powdery mildew fungi in *Arabidopsis*. During fungal infection, ABCG36 becomes concentrated at fungal entry sites, as part of the plant’s focal immune response (Stein et al., 2006; Xin et al., 2013). ABCG36 and ABCG12 are the major transporters mediating camalexin secretion in *Arabidopsis*. *abcg36/abcg12* double mutant showed greatly increased susceptibility to necrotrophic fungus *Botrytis cinerea*, and hypersensitivity to exogenous camalexin (He et al., 2019). We observed significantly reduced phosphorylation levels of ABCG36, ABCG12, ABCG19, and ABCC4 in systemic leaves of *Psm* ES4326-inoculated plants (Supplementary Table 11), suggesting that function of these proteins in SAR establishment may depend on their dephosphorylation.

### Protein Kinases

Protein kinases in plants mediate protein phosphorylation and play key roles in resistance to biological stresses (Tena et al., 2011). Kinases are usually regulated by autophosphorylation or phosphorylation by other kinases (Oh et al., 2012). We found 84 kinases showing significantly changed phosphorylation levels in systemic leaves of SAR-induced plants (Supplementary Table 6), including RLKs, CDPKs, MAPKs, and diacylglycerol kinase 7 (DGKs), indicating the broad occurrence of protein kinase phosphorylation during SAR establishment.

The innate immune systems evolved in plants are based on recognition of potential pathogens by specific pools of membrane and cytosolic receptors. In the first level of plant immune response, PAMPs (see Background) are recognized by PRRs, triggering PTI (Wu et al., 2019). PRRs comprise RLKs and receptor-like proteins (RLPs) (Wu et al., 2019). We found 17 RLKs with significantly changed phosphorylation levels in systemic leaves of *Psm* ES4326 induced plants (Supplementary Table 7). SOBIR1 (Q9SKB2) encodes an RLK that constitutively interacts with various RLPs, resulting in an RLP/SOBIR1 complex with a kinase domain that triggers downstream signaling pathways involved in plant immunity (Albert et al., 2015). SOBIR1 forms a constitutive, ligand-independent complex with leucine-rich repeat receptor protein (LRR-RP) RLP23 and Brassinosteroid Insensitive 1 (BRI1)-associated kinase (BAK1), which mediates immune activation in *Arabidopsis* (Albert et al., 2019). Paparella et al. (2014) demonstrated the importance of lysin motif-containing receptor-like kinase 3 (LYK3) in cross-talk among signaling pathways activated by ABA and pathogens. In *Arabidopsis*, LYK3 negatively regulates basal expression of defense genes and resistance to *B. cinerea* and *Pectobacterium carotovorum* infection. ABA treatment induces LYK3 expression. We identified 17 differentially phosphorylated RLKs (including RK1, CRK6, and BIR2) in addition to SOBIR1 and LYK3; these RLKs are presumably involved in phosphorylation regulation pathways in SAR.

Calcium-dependent protein kinases control a wide range of plant developmental processes, defense responses, and hormone responses (Qin et al., 2020). Nine CDPKs (CDPK1, -2, -3, -4, -5, -6, -11, -13, and -28) have been shown to play essential roles in plant immunity (Bucholc et al., 2011). Our phosphoproteome analysis identified seven CDPKs with significantly changed phosphorylation levels in SAR (Supplementary Table 7). Among these, CDPK5 and -6 (subgroup I) are redundant positive regulators of defense responses downstream of various PAMPs (e.g., flg22, elf18, PEP3, OGs), consistently with observed hypersensitivity of *cpk5* and *-6* to *P. syringae* DC3000 and *B. cinerea* (Bucholc et al., 2011; Boudsocq et al., 2012; Gravino et al., 2015). CDPK13 plays a functional role in herbivory-induced signaling networks via HsfB2a-mediated regulation of defense-related transcriptional machinery (Kanchiswamy et al., 2010). We observed significantly changed phosphorylation levels of CDPK7, -8, -21, and -32 in systemic leaves of *Psm* ES4326 locally inoculated plants, suggesting important roles of these proteins in SAR based on their phosphorylation/dephosphorylation.

### Transcription Factors

Transcription factors are important internodes in the disease resistance pathways (Hou et al., 2015), but little is known regarding whether and how their activities are mediated by protein modification. We found that at least 43 TFs were differentially phosphorylated during initial SAR induction (Supplementary Table 5), indicating that protein phosphorylation is widely involved in TF modification, and may regulate transcriptional activities of TFs during SAR. The TFS showing significantly changed phosphorylation belong to several protein families, including WRKY (WRKY6, WRKY18, WRKY40, and WRKY57), MYC (MYC2), MYB (MYB3R-5, MAMYB, and EMF), and bZIP (BZIP59 and BZIP68) (Figure 6A and Supplementary Table 5).

Non-expressor of pathogenesis-related genes 1, the SA receptor, plays key roles in plant resistance to biotic stresses. NPR1 interacts with TGA and TCP TFs in the nucleus to activate the expression of pathogenesis-related (PR) genes, including *PR1*, *PR2*, and *PR5* (Li et al., 2018). WRKY6 protein was shown by chromatin immunoprecipitation (ChIP) assay to bind directly to the NPR1 promoter containing W-box motif (Chai et al., 2014). Chen J. et al. (2019) reported that CDK8 interacts with WRKY6, WRKY18, and TGA TFs and transports RNA polymerase II to *NPR1* and *PR1* promoters and coding regions to facilitate their expression. In ETI, WRKY18 and WRKY40 act as positive regulators, as demonstrated by the strong susceptibility of *wrky18*/*wrky40* double mutant to bacterial pathogen *P. syringae* DC3000 harboring the effector *AvrRPS4* (Schön et al., 2013). Jasmonate (JA), a lipid-derived immunity hormone, promotes plant defense responses to mechanical wounding, insect attack, and pathogen infection. MYC2 plays essential roles in JA-mediated signaling pathways in plants (Wang H. et al., 2019). Guo et al. (2018) found that the stability of MYC2 was reduced when it was phosphorylated by receptor kinase FERONIA (FER). Three phosphopeptides of MYC2 showed significantly reduced abundance in systemic leaves of *Psm* ES4326 locally inoculated plants (Supplementary Table 4), suggesting that MYC2 is functionally involved in SAR establishment via dephosphorylation. Many TFs besides WRKYs and MYC2 (e.g., MYB, bZIP) are differentially phosphorylated, and are therefore promising candidate genes for studies of SAR establishment mechanism.

### Proteins Related to Plant Defense Responses

GO analysis of biological processes indicated that 22 of the 866 differentially phosphorylated proteins identified in this study are involved in plant defense responses (Figure 6B and Supplementary Table 8). MPK3 and -6, key factors in various defense responses, showed increased phosphorylation in systemic leaves of SAR-induced plants (Supplementary Table 8). MPK3/6 are involved in the activation of ethylene, camalexin, and glucosinolate biosynthetic pathways, and are essential for stomatal immunity during PTI and ETI (Devendrakumar et al., 2018). Activation by upstream MKKs in *Arabidopsis* results in phosphorylation by MPK3/6 of ACC synthase 2 (ACS2) and ACS6 at three conserved phosphorylation sites, thereby enhancing the stability of these sites and increasing ethylene biosynthesis (Liu and Zhang, 2004). MPK3/6 promote several important processes: (i) expression of ACS2 and ACS6 through phosphorylation of WRKY33 TF (Li et al., 2012); (ii) biosynthesis of glucosinolates [important secondary metabolites in plant anti-pathogen defense (Radojčić Redovniković et al., 2008)] by regulating expression of two MYB TFs involved in activation of glucosinolate biosynthesis in response to *B. cinerea* infection (Xu et al., 2016); and (iii) synthesis of SAR inducer Pip by regulating WRKY33 activity (Wang et al., 2018). MPK3/6 clearly function as carriers transmitting phosphorylation in plant immunity, but the mechanism for their phosphorylation remains unclear. Our phosphoproteomic data suggest the presence of two potential phosphorylation sites in MPK3 and one site in MPK6; this point is being addressed in ongoing studies.

Methyl salicylate, which is synthesized from SA by methyltransferases, is an essential mobile signal in plant SAR (Chen L. et al., 2019). In *Arabidopsis*, MeSA is synthesized from SA via methylation by the enzyme BSMT1 (Groux et al., 2014). During SAR induction, some SA accumulated in pathogen-infected tissues is converted to MeSA by BSMT1. MeSA is then translocated to systemic tissues via phloem or volatilization, converted back to SA by SA-binding protein (SABP2), and released SA activates systemic immune responses (Klessig et al., 2018). Thus, MeSA synthesis in pathogen-infected tissues depends on BSMT1 activity, but conversion of MeSA to SA in systemic tissues requires inhibition of BSMT1 activity. We observed significantly reduced abundance of one of the BSMT1 phosphopeptides in systemic leaves of *Psm* ES4326 locally inoculated plants (Supplementary Table 8), suggesting that BSMT1 activity is regulated by phosphorylation modification.

Differential phosphorylation occurs in many defense response-related proteins in addition to MPK3, MPK6, and BSMT1. These include cell division cycle 5 (CDC5), which is involved in plant innate immunity (Palma et al., 2007), and E3 ubiquitin ligase RING1, involved in cell death and SA-dependent defense response (Lee et al., 2011). Differential phosphorylation has also been reported for some abiotic stress-related proteins (Supplementary Table 8), e.g., enhanced response to ABA3 (ERA3), necrotic spotted lesions 1 (NSL1), cold regulated 78 (COR78), and pyrophosphorylase 6 (PPA6). Functional involvement of these proteins in responses to salt, cold, and heat stresses has been well documented, but their physiological functions during SAR are essentially unknown.

## Conclusion

**FIGURE 10:**
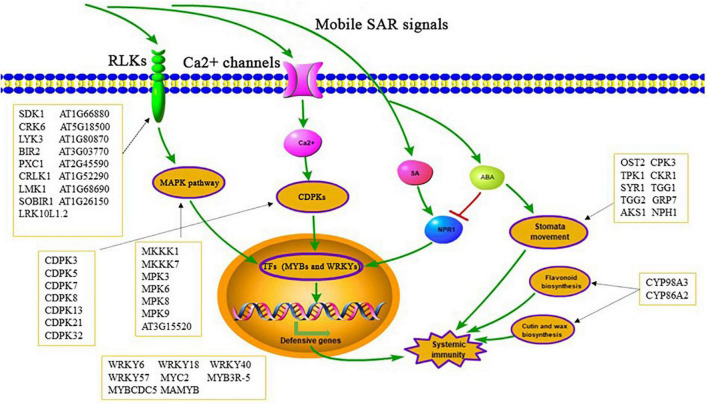
Possible pathways of significantly changed phosphoproteins involved in SAR establishment.

We identified 8011 unique phosphorylation sites from 3234 proteins in systemic leaves of *Psm* ES4326 locally inoculated and mock (MgCl_2_) treated plants. 866 significantly changed phosphoproteins, from 1119 significantly changed phosphopeptides, were detected in leaves of *Psm* ES4326-inoculated plants; these included various well-known TFs, kinases, and defense response-related proteins. In addition, we found that ABA signaling pathway and stomatal movement may be functionally involved in SAR establishment (Figure 10). Our findings indicate that SAR induction is regulated by extensive protein phosphorylation. This is the first study focused on potential SAR-related proteins using DIA quantitative phosphoproteomic analysis based on high-precision MS. Our findings help clarify phosphorylation status and sites of *Arabidopsis* proteins and will facilitate further research on the roles of phosphoproteins in SAR establishment.

## Data Availability Statement

The datasets presented in this study can be found in online repositories. The mass spectrometry proteomics data have been deposited to the ProteomeXchange Consortium (http://proteomecentral.proteomexchange.org) via the iProX partner repository with the dataset identifier PXD027512.

## Author Contributions

QZ, HG, and XH conceived and designed the experiments. QZ, QM, and XT performed the phosphoproteomics experiments. WD and KM contributed to sample collection and data analysis. QZ, HG, and ZX wrote and edited the manuscript. All authors contributed to the article and approved the submitted version.

## Conflict of Interest

WD was employed by Shanghai Omicsspace Biotechnology Co., Ltd. The remaining authors declare that the research was conducted in the absence of any commercial or financial relationships that could be construed as a potential conflict of interest.

## Publisher’s Note

All claims expressed in this article are solely those of the authors and do not necessarily represent those of their affiliated organizations, or those of the publisher, the editors and the reviewers. Any product that may be evaluated in this article, or claim that may be made by its manufacturer, is not guaranteed or endorsed by the publisher.

## Footnotes

1
https://www.genome.jp/kegg/

2
http://phosphat.uni-hohenheim.de

## Supplementary Material

Supplementary Table 1 List of DIA windows.

Supplementary Table 2 Total phosphoproteins identified in this study.

Supplementary Table 3 Identified proteins containing Tyr phosphorylation.

Supplementary Table 4 Proteins containing two or more significantly changed phosphopeptides.

Supplementary Table 5 Differentially phosphorylated TFs in SL-V vs. SL-CK.

Supplementary Table 6 Differentially phosphorylated kinases in SL-V vs. SL-CK.

Supplementary Table 7 Differentially phosphorylated RLKs, CDPKs, and MAPKs.

Supplementary Table 8 Differentially phosphorylated defense-related proteins and abiotic stress-related proteins.

Supplementary Table 9 Comparative analyses of phosphoproteome data set related to stomata immunity and the current study.

Supplementary Table 10 Differentially phosphorylated proteins related to SA and ABA signaling pathway.

Supplementary Table 11 Differentially phosphorylated ABC transporter family proteins.

Supplementary Table 12 Primers used for RT-qPCR.

Supplementary Data 1 List of representative MS2 data.
