# Supplementary material for: Protein Phosphorylation Changes During Systemic Acquired Resistance in Arabidopsis thaliana
Source: Front Plant Sci. 2021 Nov 11;12:748287. doi: 10.3389/fpls.2021.748287 (PMC8632492; doi:10.3389/fpls.2021.748287)
Supplement: Supplementary Table 12 — Primers used for RT-qPCR. [file Table_12.DOCX]

Primer sequences used in this study.

| **RT-qPCR analysis** | | |
| --- | --- | --- |
| AT5G53830 | ACGCCACTGAGAAGCAACGATG | Forward |
|  | TCTAGGAGTTGAGACTGGAGATGGATG | Reverse |
| AT5G55660 | TCTGGTTACAAATGGCAAGGAGATGAG | Forward |
|  | TGTGGTAGCCTTAGCAACTGATATGTC | Reverse |
| AT5G57580 | GCACAGAGGTATGGTGAAGTAGAAGG | Forward |
|  | AACGAGCAGAAGAAGGCGAAGATG | Reverse |
| AT5G57610 | GCACAGAGGTATGGTGAAGTAGAAGG | Forward |
|  | AACGAGCAGAAGAAGGCGAAGATG | Reverse |
| AT5G57870 | CTGTTTGGTGAGGAAGGGACTTGG | Forward |
|  | AGAGAAGCGACTTGCGGATTGAAC | Reverse |
| AT5G60410 | GAGGTGGAGATGATAATGCCGACAG | Forward |
|  | CCGCTCATAGGACACCGAAGATTG | Reverse |
| AT5G61210 | GCAAATCTTCCCAAGCATAACTCAGTC | Forward |
|  | CATATCAGCCAAAGAGGGTTCAGAGG | Reverse |
| AT5G62220 | CTCCACCACCTCCATTGATCGTAAG | Forward |
|  | GAAGCGGCGATGTAGGAAGAGC | Reverse |
| PR1 | GCT CTT GTA GGT GCT CTT GTT C | Forward |
|  | GCC TCT TAG TTG TTC TGC GTA G | Reverse |
| ACTIN8 | TGTGCCTATCTACGAGGGTTT | Forward |
|  | TTTCCCGTTCTGCTGTTGT | Reverse |
