# Supplementary material for: Protein Phosphorylation Changes During Systemic Acquired Resistance in Arabidopsis thaliana
Source: Front Plant Sci. 2021 Nov 11;12:748287. doi: 10.3389/fpls.2021.748287 (PMC8632492; doi:10.3389/fpls.2021.748287)
Supplement: Supplementary Data 1 — List of representative MS2 data. [file Data_Sheet_1.docx]

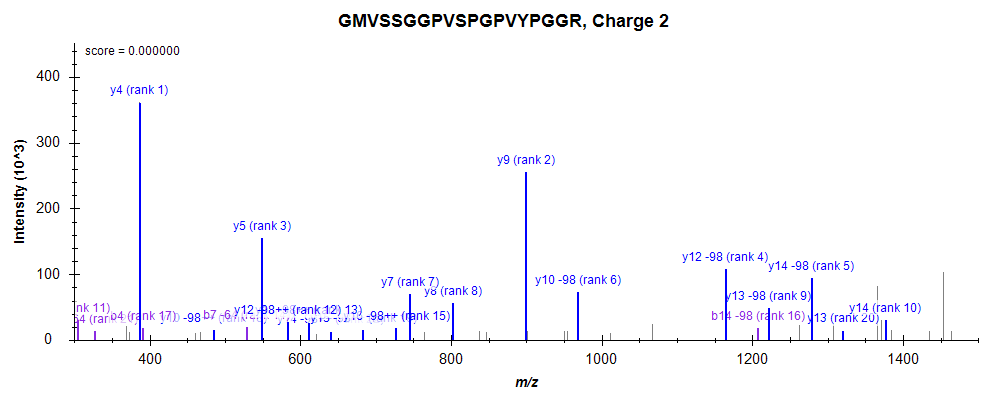


GM[Oxidation]VSSGGPVS[Phospho]PGPVYPGGR


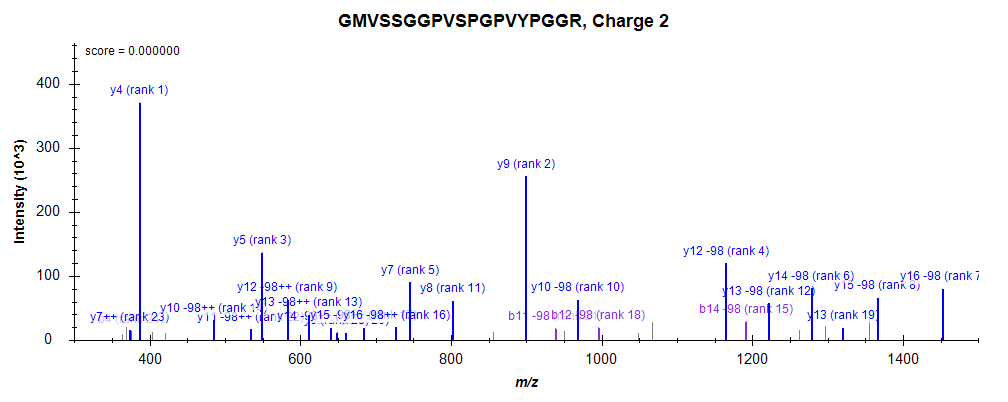


GMVSSGGPVS[Phospho]PGPVYPGGR


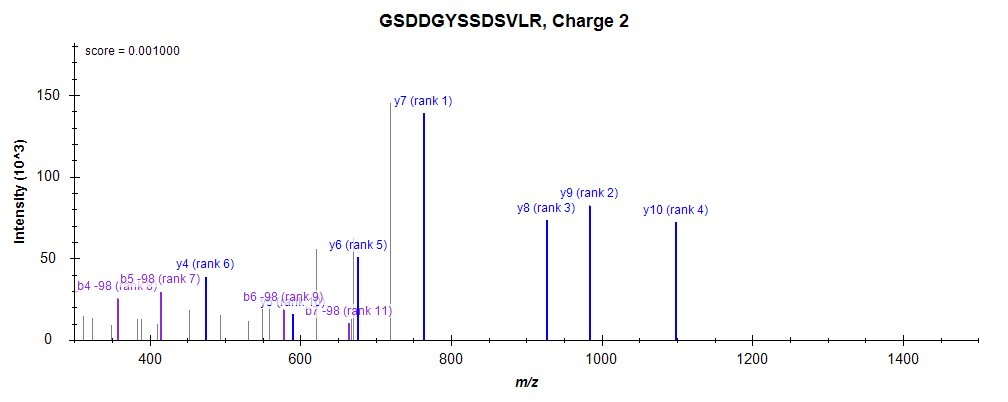


GS[Phospho]DDGYSSDSVLR


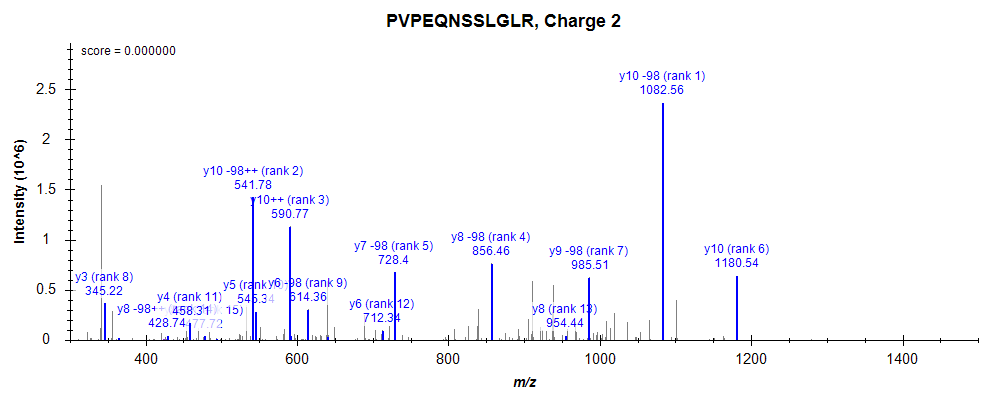


PVPEQNS[Phospho]S[Phospho]LGLR


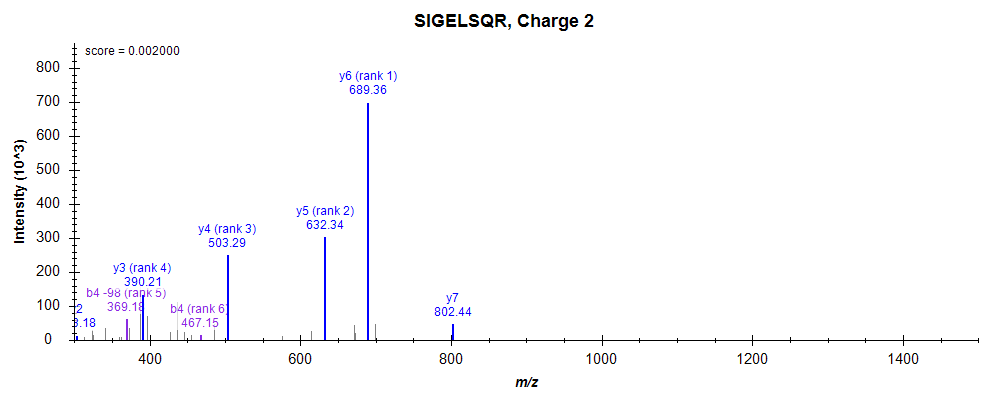


S[Phospho]IGELSQR


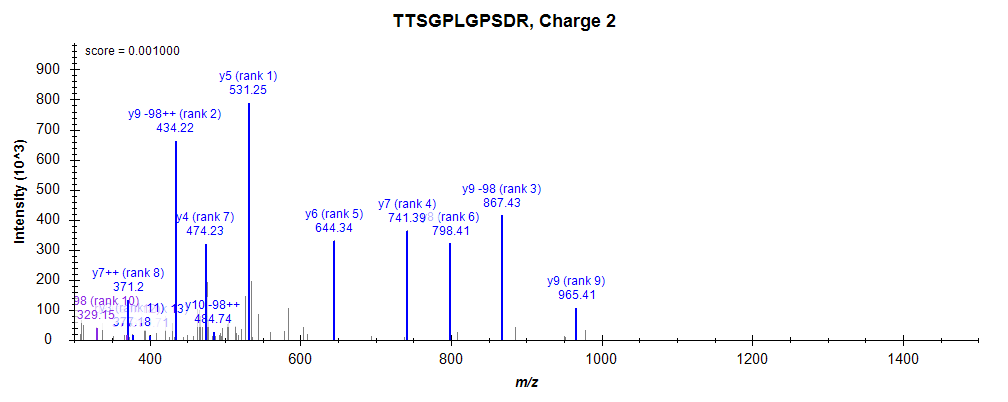


T[Phospho]TS[Phospho]GPLGPSDR


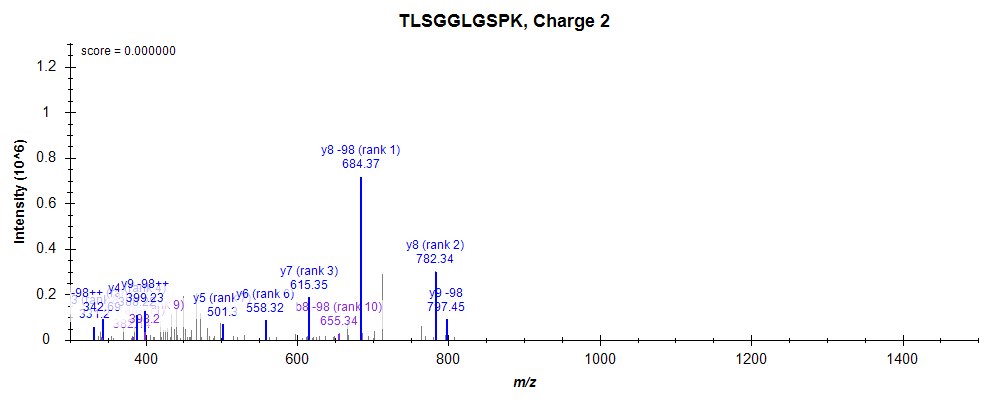


TLS[Phospho]GGLGS[Phospho]PK


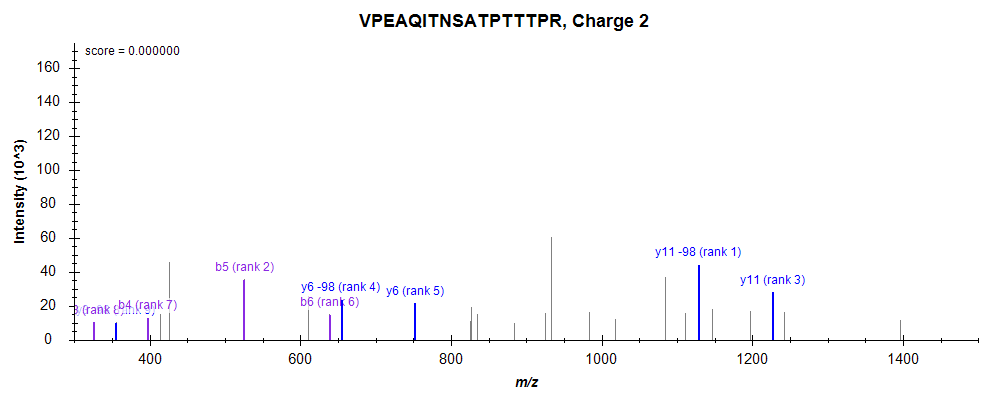


VPEAQITNSATPTTT[Phospho]PR


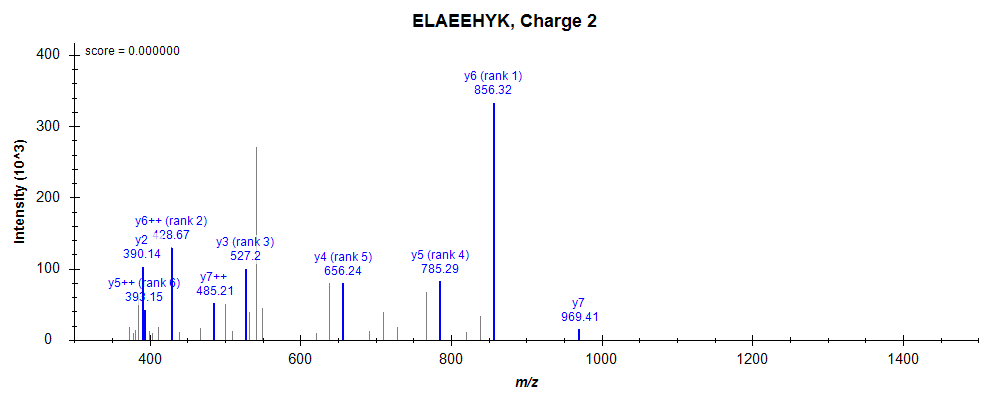


ELAEEHY[Phospho]K


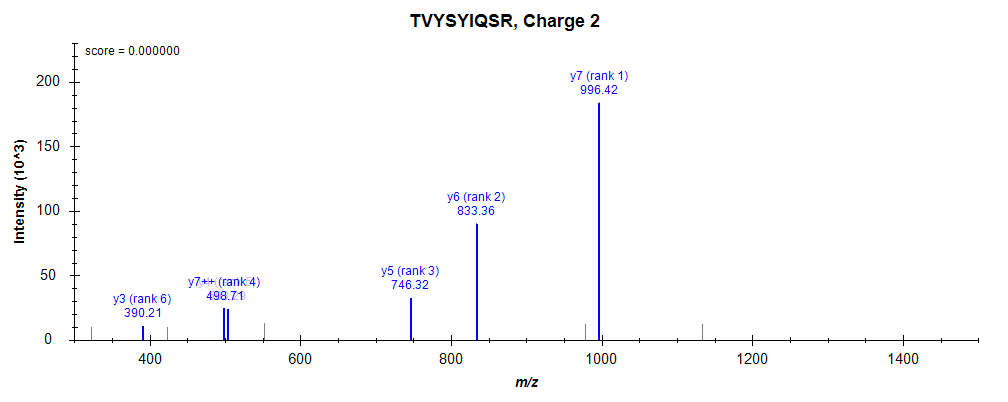


TVYSY[Phospho]IQSR


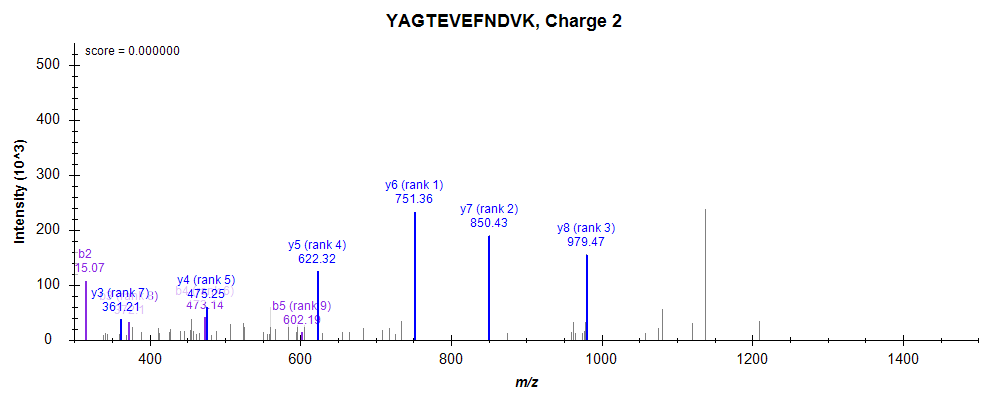


Y[Phospho]AGTEVEFNDVK


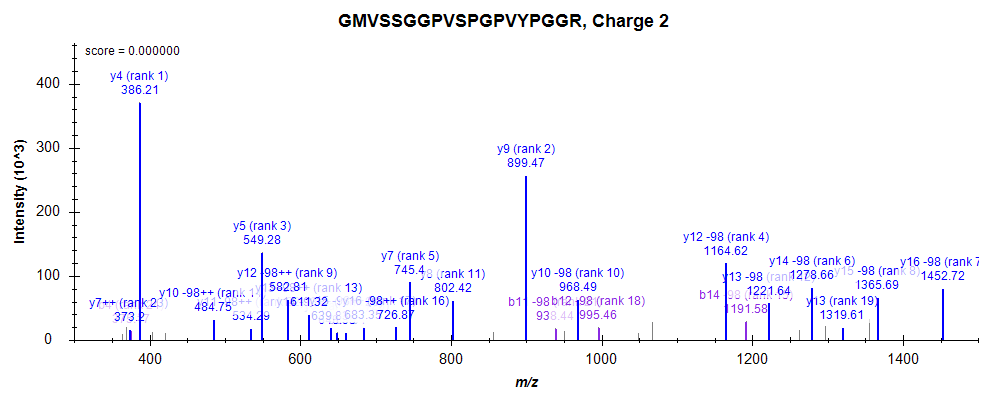


GMVSSGGPVS[Phospho]PGPVYPGGR


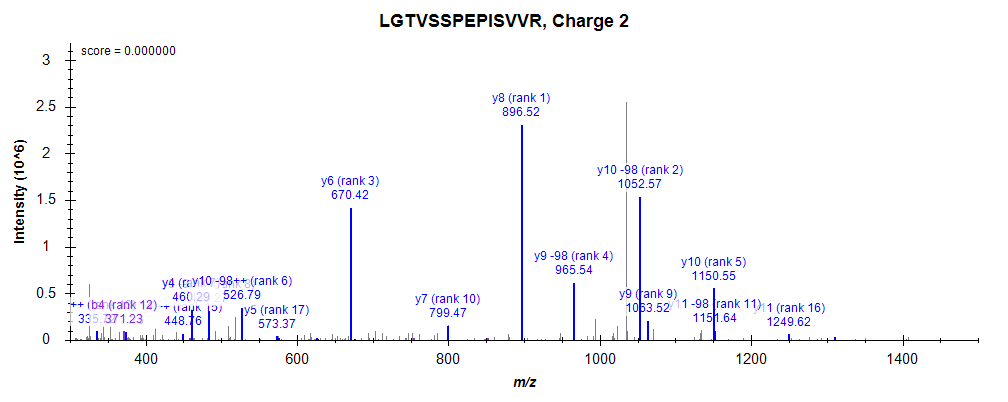


LGTVSS[Phospho]PEPISVVR


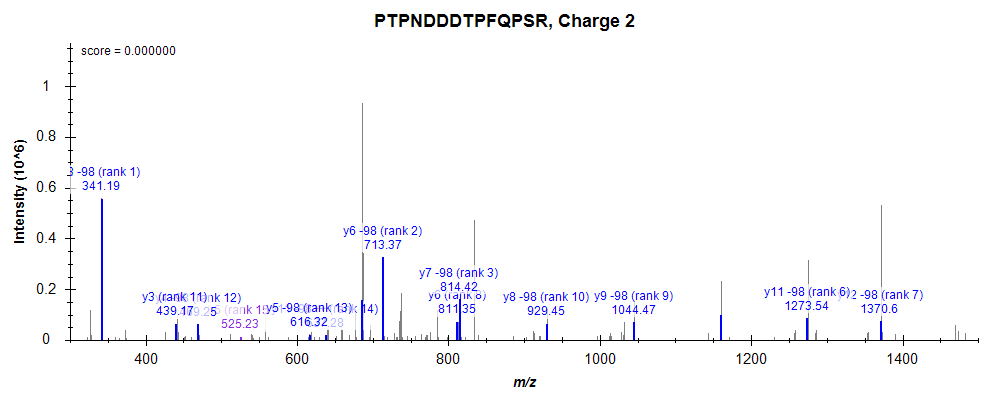


PTPNDDDTPFQPS[Phospho]R


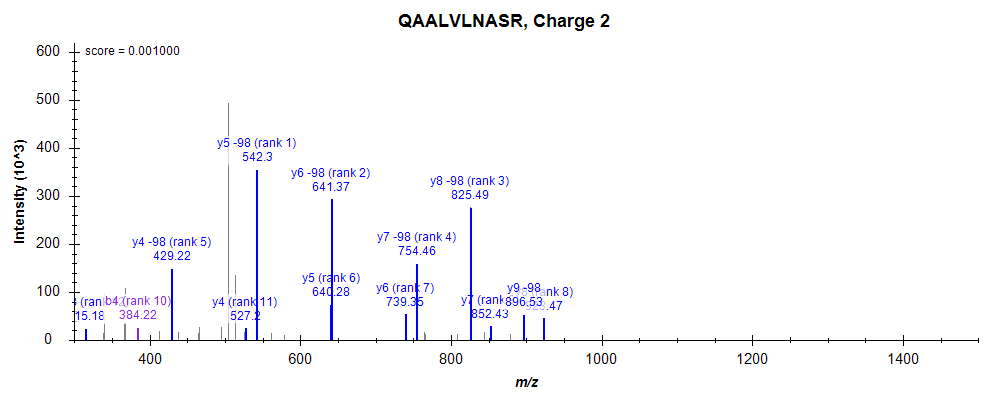


QAALVLNAS[Phospho]R
